# Cross-neutralization activity against SARS-CoV-2 is present in currently available intravenous immunoglobulins

**DOI:** 10.2217/imt-2020-0220

**Published:** 2020-09-09

**Authors:** José María Díez, Carolina Romero, Júlia Vergara-Alert, Melissa Belló-Perez, Jordi Rodon, José Manuel Honrubia, Joaquim Segalés, Isabel Sola, Luis Enjuanes, Rodrigo Gajardo

**Affiliations:** ^1^Bioscience Research & Development, Grifols, Barcelona, Spain; ^2^IRTA, Centre de Recerca en Sanitat Animal (CReSA, IRTA-UAB), Campus de la Universitat Autònoma de Barcelona (UAB), Bellaterra, Barcelona, Spain; ^3^Laboratorio Coronavirus. Departamento de Biología Molecular y Celular, CNB-CSIC, Madrid, Spain; ^4^UAB, CReSA (IRTA-UAB), Campus de la Universitat Autònoma de Barcelona (UAB), Bellaterra, Barcelona, Spain; ^5^Departament de Sanitat i Anatomia Animals, Facultat de Veterinària, Universitat Autònoma de Barcelona (UAB), Bellaterra, Barcelona, Spain

**Keywords:** COVID-19, cross-neutralization, intravenous immunoglobulin, MERS-CoV, SARS-CoV, SARS-CoV-2

## Abstract

**Background:** Cross-reactivity against human coronaviruses with Flebogamma^®^ DIF and Gamunex^®^-C, two available intravenous immunoglobulins (IVIG), has been reported. In this study, these IVIG were tested for neutralization activity against severe acute respiratory syndrome coronavirus 2 (SARS-CoV-2), SARS-CoV and Middle East respiratory syndrome CoV (MERS-CoV). **Materials & methods:** Neutralization capacity of lots of IVIG manufactured prior to COVID-19 pandemic was assessed against these viruses in cell culture. Infectivity neutralization was quantified by percent reduction in plaque-forming units and/or cytopathic/cytotoxic methods. **Results:** All IVIG preparations showed neutralization of SARS-CoV-2 isolates. All IVIG lots produced neutralization of SARS-CoV. No IVIG preparation showed significant neutralizing activity against MERS-CoV. **Conclusion:** The tested IVIG contain antibodies with significant *in vitro* cross-neutralization capacity against SARS-CoV-2 and SARS-CoV, but not MERS-CoV. These preparations are currently under evaluation as potential therapies for COVID-19.

The outbreak of the novel severe acute respiratory syndrome coronavirus 2 (SARS-CoV-2) which causes the respiratory disease COVID-19 was declared a pandemic by the WHO in March 2020. Most infected patients (80%) have mild symptoms. However, about 20% of COVID-19 patients can progress to severe pneumonia and to acute respiratory distress syndrome which is associated with multi-organ failure and death [1]. The current critical situation demands an effective and reliable therapy that is immediately available to control the progression of the disease [2]. Convalescent plasma or plasma-derived immunoglobulin (IG; either polyvalent IG prepared from healthy donors or hyperimmune IG prepared from donors with high antibody titers against a specific antigen) have been historically used as a readily available therapeutic option in outbreaks of emerging or re-emerging infections [3].

To date, seven human coronaviruses (HCoV) have been identified. Four of them (HCoV-229E, HCoV-NL63, HCoV-OC43 and HCoV-HKU1) are globally distributed [4] and are associated with about 15% of common colds, typically causing mild symptoms [5]. In contrast, SARS-CoV, Middle East respiratory syndrome CoV (MERS-CoV), and SARS-CoV-2 are zoonotic epidemic viruses [6] that can cause severe respiratory infections and fatalities. SARS-CoV emerged in China in 2002 with the last reported case in 2014. MERS-CoV emerged in Saudi Arabia a decade later, in 2012, and led to an outbreak in South Korea in 2015. MERS-CoV still emerges sporadically in humans from its reservoir in camelids [7–9]. More recently (December 2019), the novel coronavirus SARS-CoV-2 emerged in China and because of its extraordinary human-to-human transmissibility is currently causing an unprecedented pandemic [10].

Coronaviruses share some morphological and functional properties that may be associated with cross-reactive immune responses which may have important therapeutic implications [11]. SARS-CoV, SARS-CoV-2 and MERS-CoV are classified within the family *Coronaviridae*, genus *Betacoronavirus*, subgenera *Sarbecovirus* (SARS-CoV, SARS-CoV-2) and *Merbecovirus* (MERS-CoV). SARS-CoV-2 has four main structural proteins including spike (S) glycoprotein, small envelope (E) glycoprotein, membrane (M) glycoprotein and nucleocapsid (N) protein [12]. S protein is the main determinant of the coronavirus entry into the host cell and is also the major target of neutralizing antibodies [13,14]. Spikes are formed by trimers of protein S, which is in turn formed by subunit (S1) that mediates the binding to the cell receptor, and a membrane-anchored subunit (S2) that mediates the fusion of the virus with cell membranes [15]. The receptor-binding domain (RBD) is a key functional component within the S1 subunit that is responsible for virus binding to host cell [16]. Potent neutralizing antibodies often target RBD. However, the S1 subunit shows a higher variability than S2. Antibodies targeting S1 are often virus-specific making S2 a better target for cross-neutralizing antibodies [17,18].

The amino-acid sequence identity among the S proteins of human betacoronaviruses causing mild (HCoV-OC43 and HCoV-HKU1) and severe (SARS-CoV, SARS-CoV-2 and MERS-CoV) respiratory infections varies between 22 and 33% [14]. However, the S proteins of SARS-CoV and SARS-CoV-2 share 77% amino-acid identity [19]. Shared protein homologies among coronaviruses can cause cross-reactivity and/or cross-neutralization antigenic responses (i.e., antibodies able to recognize a coronavirus, but that have generated in response to prior infection of other different circulating coronavirues). Cross-reactivity has been described among HCoVs of the same genus, particularly betacoronaviruses. Cross-reactivity between SARS-CoV, MERS-CoV and other endemic HCoVs has been reported in some studies [20–22]. However, it is uncertain whether such cross-reacting antibodies among coronaviruses have also the capacity of reducing viral infectivity by a cross-neutralization effect.

Recently, we reported cross-reactivity in ELISA binding assays against antigens of SARS-CoV, SARS-CoV-2 and MERS-CoV with Flebogamma^®^ DIF 5 and 10% and Gamunex^®^-C, two currently available intravenous IGs (IVIG) [23]. As a continuation of this study, here we evaluated the neutralization capacity of those same IVIG products against these epidemic HCoVs.

## Material & methods

### Experimental products

IVIG products used in this study were Flebogamma^®^ DIF 5% and 10% (Instituto Grifols S.A., Barcelona, Spain) and Gamunex^®^-C 10% (Grifols Therapeutics Inc., NC, USA), two highly purified (≥98–99% immunoglobuin G [IgG]), unmodified human IGs. Each product is manufactured from plasma collected from thousands of donors in the USA and/or several European countries. IgG concentrations in Flebogamma DIF products were 50 and 100 mg/ml (5 and 10%) and in Gamunex-C, the concentration was 100 mg/ml (10%). To ensure a virus-free product, both IVIG manufacturing processes contain dedicated steps with high pathogen clearance capacity, such as solvent/detergent treatment, heat treatment, caprylate treatment and nanofiltration (Planova™, Asahi Kasei, Brussels, Belgium). The plasma used to manufacture the IVIG lots tested was collected from March 2018 to October 2019.

### Study design

Six different lots of Flebogamma DIF and Gamunex-C were tested at several dilutions for cross-reactivity against SARS-CoV, SARS-CoV-2 and MERS-CoV by: ELISA techniques; and well-established neutralization assays in cell cultures. Lots were identified as F1 and F2 for Flebogamma 5% DIF, F3 and F4 for Flebogamma 10% DIF and G1 and G2 for Gamunex-C. Each experiment was performed in duplicate.

Handling of viruses and cell cultures was carried out at the Level 3 Biosafety Laboratories in the *Centro Nacional de Biotecnología – Consejo Superior de Investigaciones Científicas* (CNB-CSIC; Madrid, Spain) and the *Institut de Recerca i Tecnologia Agroalimentàries – Centre de Recerca en Sanitat Animal* (IRTA-CReSA; Barcelona, Spain), following the centers’ biohazard safety guidelines and under authorizations #A/ES/00/I-8 and #SA-10430-20, respectively.

### Virus strains

Recombinant SARS-CoV was generated from Urbani strain using a previously described reverse genetic technique [24]. Two different SARS-CoV-2 isolates, collected from nasopharyngeal swab from COVID-19 patients, were tested: SARS-CoV-2 MAD6 isolated from a 69-year-old male patient from Madrid (Spain); and SARS-CoV-2 (accession ID EPI_ISL_418268 at GISAID repository: http://gisaid.org) isolated from a an 89-year-old male patient from Badalona (Spain). Both stock viruses were prepared by collecting the supernatant from Vero E6 cells, as previously described [25]. Recombinant MERS-CoV was generated using a previously described reverse genetic system [26] from the reference sequence of MERS-CoV isolated from the index patient EMC/2012 (GeneBank JX869059) [27].

### Cell lines & culture

Huh7 is a well differentiated human hepatocyte-derived carcinoma cell line, kindly provided by Dr L Carrasco (Centr*o de Biología Molecular Severo Ochoa – Consejo Superior de Investigaciones Científicas* [CBMSO-CSIC], Madrid, Spain). Huh7 is composed of epithelial-like cells susceptible to infection by MERS-CoV [28].

Vero E6 is a cell line isolated from kidney epithelial cells extracted from an African green monkey. Vero E6 is composed of epithelial-like cells susceptible to infection by SARS-CoV and SARS-CoV-2 [29].

At CNB-CSIC, Vero E6 cell lines were kindly provided by Dr E Snjider (University of Leiden Medical Center, The Netherlands). Both Huh7 and Vero E6 cell lines were cultured in Dulbecco’s modified Eagle medium (DMEM) supplemented with 25 mM HEPES buffer, 2 mM l-glutamine (Sigma-Aldrich, MI, USA), 1% nonessential amino-acids (Sigma-Aldrich), 10% fetal bovine serum (FBS; BioWhittaker, Inc., MD, USA). In the post infection semisolid medium, the percentage of FBS was reduced to 2%, and diethylaminoethyl (DEAE)-dextran (Sigma-Aldrich) was added to a final concentration of 0.08 mg/ml.

At IRTA-CReSA, Vero E6 cells were obtained from the ATCC (ATCC CRL-1586) and cultured in DMEM (Lonza, Basel, Switzerland) supplemented with 5% FBS (EuroClone, Pero, Italy), 100 U/ml penicillin, 100 μg/ml streptomycin and 2 mM glutamine 8 (all from ThermoFisher Scientific, MA, USA). In the post infection medium, the percentage of FBS was 2%.

### IgG ELISA testing procedures

Qualitative determination of IgG class antibodies cross-reactivity against antigens of the tested coronaviruses was performed using ELISA techniques. IVIG samples were serially diluted using the buffer solutions provided in each IgG ELISA kit. The following kits were used for the qualitative determination of IgG class antibodies in the experimental IVIG lots: SARS Coronavirus IgG ELISA kit (Creative Diagnostics, NY, USA), against virus lysate; Human Anti-SARS-CoV-2 Virus Spike 1 [S1] IgG ELISA Kit (Alpha Diagnostic Intl. Inc., TX, USA), against S1 subunit spike protein; RV-402100-1, Human Anti-MERS-NP IgG ELISA Kit (Alpha Diagnostic Intl Inc.), against N protein; RV-402400-1, Human Anti-MERS-RBD IgG ELISA Kit (Alpha Diagnostic Intl Inc.), against RBD of S1 subunit spike protein (S1/RBD); RV-402300-1, Human Anti-MERS-S2 IgG ELISA Kit (Alpha Diagnostic Intl. Inc.), against S2 subunit spike protein; RV-405200 (formerly RV-404100-1). In all cases, the determinations were carried out following the manufacturer’s instructions. Reactivity was rated as negative if no reaction was observed with neat IVIG or positive if the lowest IVIG dilution demonstrated reactivity.

### Neutralization assay for SARS-CoV, SARS-CoV-2 (MAD6 isolate) & MERS-CoV

This neutralization assay was based on the reduction in plaque forming units (PFU) after exposing a given amount of virus to the product to be characterized and comparing with the untreated control. This assay is performed in cell cultured plates with a semisolid ovarlay to allow plaque formation. For this assay, IVIG samples were serially diluted (factor 10 dilutions: 1:10^2^, 1:10^3^, 1:10^4^ and 1:10^5^) in Dulbecco’s phosphate-buffered saline (Gibco, Thermo Fisher Scientific, MA, USA). Samples of each IVIG dilution were incubated for 1 h (37°C; 5% CO_2_) with 300 PFUs of SARS-CoV, SARS-CoV-2 or MERS-CoV. Aliquots of 50 μl of each IVIG dilution-virus complex were added in duplicate to confluent monolayers of Vero E6 cells (for SARS-CoV and SARS-CoV-2) or Huh7 (for MERS-CoV), seeded in 12-well plates and incubated for 1 h (37°C; 5% CO_2_). After this adsorption time, the IgG-virus complex inoculum was removed and a semi-solid overlay was added (DMEM 2% FBS + 0.6% agarose). Cells were incubated for 72 h at 37°C. The semi-solid medium was removed, the cells were fixed with 10% neutral buffered formaldehyde (Sigma-Aldrich) for 1 h at room temperature, and stained with 0.2% aqueous gentian violet for 10 min, followed by plaque counting. The sensitivity threshold of the technique was 20 PFU per ml.

The neutralization potency of the IVIG products was expressed in two ways: percent reduction in PFU calculated from the PFU count after neutralization by IVIG relative to initial PFU count inoculated onto the cells; and plaque reduction neutralization test (PRNT_50_) value, calculated as the -log_10_ of the reciprocal of the highest IVIG dilution to reduce the number of plaques by 50% compared with the number of plaques without IVIG.

### Neutralization assay for SARS-CoV-2 (EPI_ISL_418268 isolate)

This neutralization assay measured the cytopathic/cytotoxic virus-induced effect by detecting cellular enzymatic activity after incubation with a given amount of the relevant virus and comparing this with the relevant untreated control. For this assay, a fixed concentration of a SARS-CoV-2 stock (10^1.8^ TCID_50_/ml, a concentration that achieves 50% cytopathic effect) was mixed with decreasing concentrations of the IVIG samples (range 1:10 to 1:5120), each mixture was incubated for 1 h at 37°C, and added to Vero E6 cells. To assess potential plasma-induced cytotoxicity, Vero E6 cells were also cultured with the same decreasing concentrations of plasma in the absence of SARS-CoV-2. Uninfected cells and untreated virus-infected cells were used as negative and positive infection controls, respectively (see Supplementary Figure 1). Plasma from a COVID-19 positive patient with a high half-maximal inhibitory concentration (IC_50_) was included as an active positive control (expressed as the -log_10_ of the reciprocal of the dilution). All the cultures were incubated at 37°C and 5% CO_2_ for 3 days.

Cytopathic or cytotoxic effects of the virus or plasma samples were measured at 3 days post-infection, using the Cell Titer-Glo luminescent cell viability assay (Promega, WI, USA). Luminescence was measured in a Fluoroskan Ascent FL luminometer (Thermo Fisher Scientific). Neutralization curves are shown as nonlinear regressions. IC_50_ values were determined from the fitted curves as the plasma dilutions that produced 50% neutralization. Details of the technique are available elsewhere [25].

## Results

### Cross-reactivity studies (ELISA-binding assays)

IVIG products showed consistent reactivity to antigens of SARS-CoV (culture lysate) at 10–100 mg/ml IgG, SARS-CoV-2 (S1 subunit protein) at 100 μg/ml IgG and MERS-CoV (N protein, S1 subunit/RHD protein and S2 subunit protein) at 50 μg/ml IgG (Table 1).

**Table 1.** Intravenous immunoglobulin reactivity against severe acute respiratory syndrome coronavirus (SARS-CoV), severe acute respiratory syndrome coronavirus 2 (SARS-CoV-2) and Middle East respiratory syndrome coronavirus (MERS-CoV).

IVIG product (lots)	% IgG	Country of origin of the plasma	Virus and antigen/target				
			SARS-CoV Culture lysate (mg/ml)	SARS-CoV-2 S1 subunit (µg/ml)	MERS-CoV		
					N protein (µg/ml)	S1 subunit/RBD (µg/ml)	S2 subunit (µg/ml)
F1	5	Germany	50	100	50	50	50
F2	5	Czech Republic	10	100	50	50	50
F3	10	USA	100	100	50	50	50
F4	10	Spain	100	100	50	50	50
G1	10	USA	100	100	50	50	50
G2	10	USA	100	100	50	50	50

Two independent assays were performed on each IVIG product lot by ELISA. Concentration denotes the least potent IVIG dilution with positive result. IgG: Immunoglobulin G; IVIG: Intravenous immunoglobulin.

### Neutralization studies of SARS-CoV

**Figure 1.**
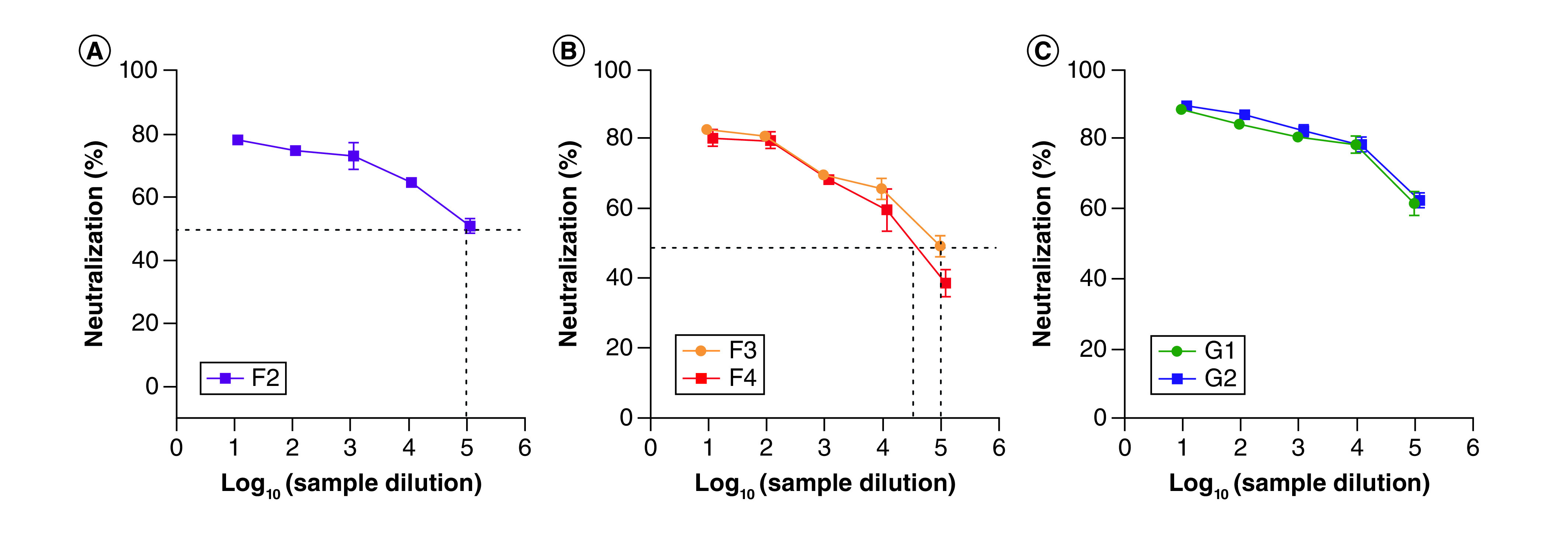
Percentage of cross-neutralization capacity of intravenous immunoglobulin against severe acute respiratory syndrome coronavirus calculated from reduction of plaque forming unit counts versus serial dilutions. Dotted lines indicate the PRNT_50_ values. **(A)** Neutralization by lots F1 and F2 lots of 5% intravenous immunoglobulin (IVIG); **(B)** neutralization by lots F3 and F4 lots of 10% IVIG; **(C)** neutralization by lots G1 and G2 10% IVIG.

All the assayed IVIG preparations had neutralizing activity against SARS-CoV ranging from 39 to 61% (Figure 1). All 10% IgG IVIG preparations (F3, F4, G1 and G2) showed PRNT_50_ neutralization titers between 2.0 and 3.3, corresponding to 50–61% PFU reduction (Figure 1B & C). The highest PFU reductions, 59.3 and 61.9% (PRNT_50_ neutralization titers of 3.2 and 3.3), were observed with lots F4 and G1, respectively, at 1 and 0.1 mg/ml IgG (dilution factors 2 and 3). The F1 and F2 lots, (5% IgG) showed a lower neutralization capacity with PFU reductions of 39.5 and 43.3%, respectively (Figure 1A).

### Neutralization studies of SARS-CoV-2

**Figure 2.**
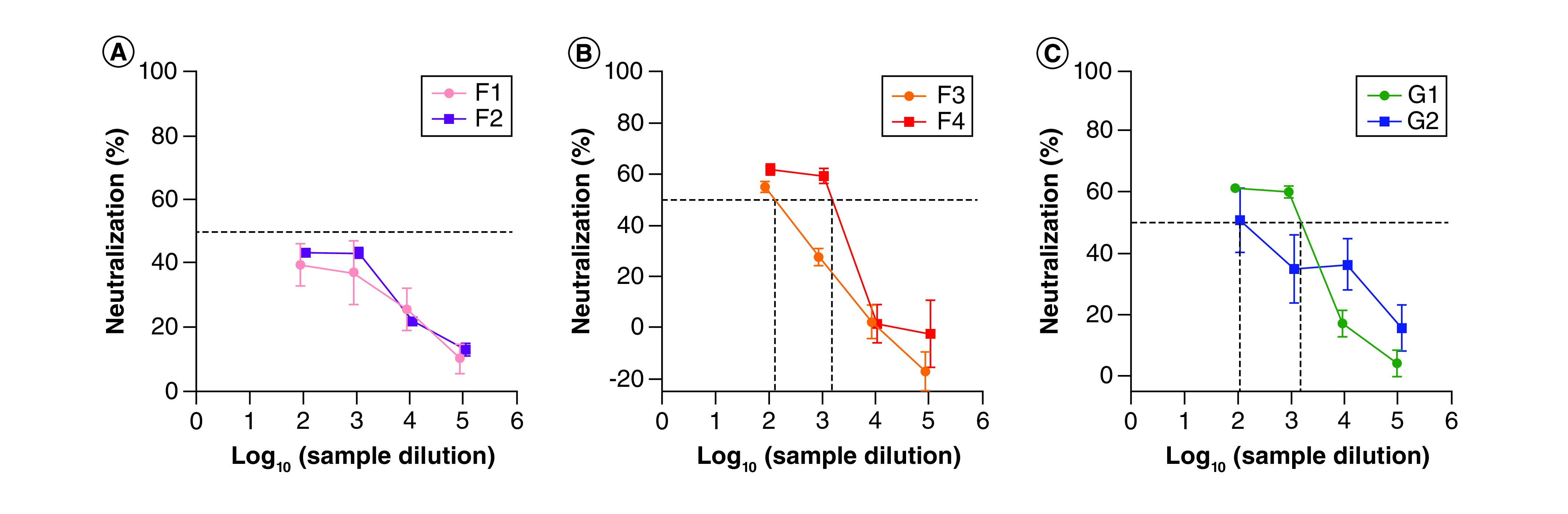
Percentage of cross-neutralization capacity of intravenous immunoglobulin against severe acute respiratory syndrome coronavirus 2 (MAD6 isolate) calculated by reduction of plaque forming units versus serial dilutions. Dotted lines indicate the PRNT_50_ values. **(A)** Neutralization by lot F2 5% intravenous immunoglobulin (IVIG); **(B)** neutralization by lots F3 and F4 of 10% IVIG; **(C)** neutralization by lots G1 and G2 of 10% IVIG.

For SARS-CoV-2 MAD6 isolate, all IVIG lots, except F1 (inconclusive results) showed a significant neutralizing activity and reached PRNT_50_ titers ranging from 4.5 to >5 (Figure 2). PFU reductions ranging from 78.2 to 82.5% were observed with lots F2, F3 and F4 at a dilution factor of one. Even at the highest dilution factor (5 = 0.5 and 1 μg/ml), the PFU reduction ranged from 38.5 to 50.9% corresponding to PRNT_50_ titers of 4.5–5.0 (Figure 2A & B). For lots G1 and G2, the PFU reduction was even higher, ranging from 88.5 to 89.5% at a dilution factor of one to 61.7–62.5% at a dilution factor of five with PRNT_50_ titers greater than five (Figure 2C).

**Figure 3.**
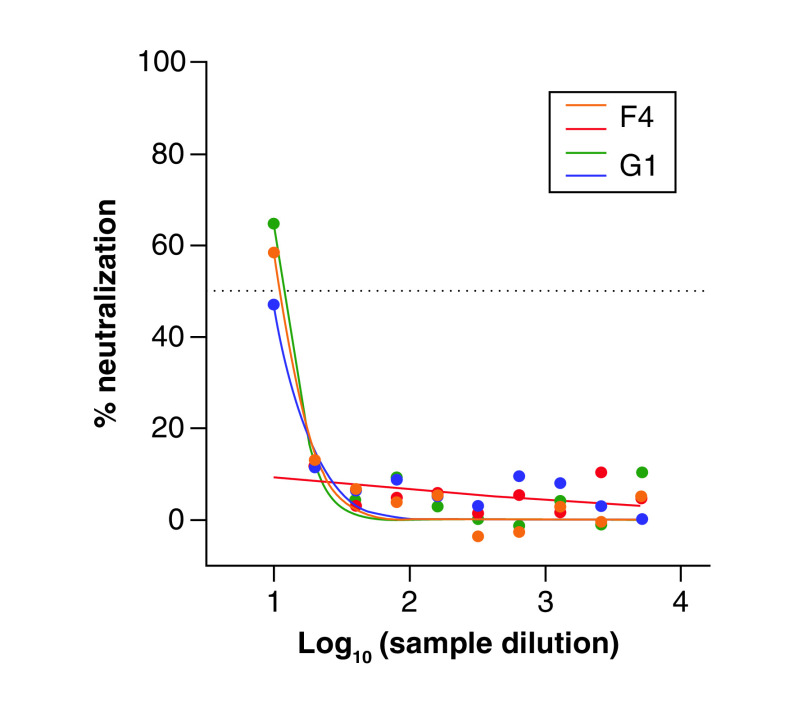
Percentage of cross-neutralization capacity of intravenous immunoglobulin lots (F4: 10% intravenous immunoglobulin; G1: 10% intravenous immunoglobulin) against severe acute respiratory syndrome coronavirus 2 (EPI_ISL_418268 isolate) calculated by reduction of cytopathic effect versus serial dilutions.

For the SARS-CoV-2 EPI_ISL_418268 isolate, F4 and G1 lots neutralized 58.4 and 64.7%, respectively, TCID_50_ counts at a dilution factor of one (Figure 3). One replicate of F4 product failed to demonstrate neutralization.

### Neutralization studies of MERS-CoV

No IVIG lot showed any significant PFU reduction (i.e., >10%) on MERS-CoV even at the lowest dilution factor (10 mg/ml IgG).

## Discussion

The results presented here demonstrate for the first time significant cross-neutralization activity against SARS-CoV and especially SARS-CoV-2 in two therapeutic IVIG concentrates (Flebogamma^®^ DIF and Gamunex^®^-C). This neutralizing activity correlates with the cross-reactivity to different coronavirus antigens observed in ELISA-binding assays with IVIG, as shown in a previous study [23]. The plasma used to manufacture the tested IVIG lots was collected prior the detection of SARS-CoV-2 in Europe and the USA. Therefore, these results should be ascribed to cross-reactivity against SARS-CoV-2 by antibodies against endemic HCoVs in the human population at large. Similar results have been reported for SARS-CoV and MERS-CoV [20–22].

IVIG are polyclonal IgG antibodies reacting to a broad range of different antigens. Antibody titers and specificities may vary slightly among different lots and manufacturers, depending on the plasma donor population [30]. Our neutralization studies showed that the studied IVIG products contain antibodies with cross-neutralizing capacity against SARS-CoV (40–60%) and SARS-CoV-2 (80–90%), but not against MERS-CoV (<10%). These results suggest that the cross-neutralizing antibodies target antigenic regions more conserved in SARS-CoV and SARS-CoV-2 than in MERS-CoV.

No significant differences in the neutralizing capacity were observed among IVIG lots regardless the country of origin for the plasma. This reinforces the broad applicability of these results. Two different neutralization techniques were used for SARS-CoV-2 and both techniques showed not only the IVIG neutralization capacity, but also the reliability of the results. In addition, results obtained with two different SARS-CoV-2 isolates confirm that the neutralization capacity is not dependent on the isolate. This was not unexpected since no significant sequence differences have been observed among SARS-CoV-2 isolates currently circulating throughout the world.

The percentage of SARS-CoV-2 cross-neutralization was higher in the PFU reduction technique than in the cytopathic effect/cytotoxic technique with very low or negative values in some few cases (inconclusive for lot F1 by the PFU study and cytopathic effect in one replicate of lot F4). This suggests that the technique used and/or slight variations in methodology may significantly influence the nature or magnitude of the results. Therefore, further evaluation this cross-neutralizing activity should be carried out.

Cross-neutralization is gaining attention as a protective mechanism against viral infection in the context of the COVID-19 health emergency. The results of this study are in agreement with recent studies that describe cross-neutralization of SARS-CoV-2 by monoclonal antibodies from memory B cells of an individual who was infected with SARS-CoV [31]. Furthermore, SARS-CoV-2-reactive CD4^+^ T cells have been detected in around half of unexposed individuals, suggesting that there is cross-reactive T-cell recognition between circulating common cold coronaviruses and SARS-CoV-2 [32]. However, the levels of cross-neutralizing antibodies against SARS-CoV-2 in the sera of SARS-CoV patients can be highly variable [33]. IVIG products are prepared using plasma from thousands of different donors, hence containing a broad representation of the state of immunity in the population at that time. This is consistent with the low rate of variability found among the different lots of IVIG products tested. Nevertheless, greater variability is expected among individuals with respect to infection by a given endemic human coronavirus. Therefore, it has been hypothesized that the diversity of symptoms observed in SARS-CoV-2-infected individuals and even the potential for getting infected may depend on pre-existing cross-immunity due to previous exposure to other endemic HCoVs. In this regard, a detailed study of the state of immunity in the general population distinguishing those affected and not affected by the SARS-CoV-2 may be warranted.

The higher cross-neutralizing capacity of the tested IVIG preparations against SARS-CoV and SARS-CoV-2 than MERS-CoV may be explained by higher sequence identity of the S proteins of circulating mild HCoVs (HCoV-OC43 and HCoV-HKU1) with SARS-CoV and SARS-CoV-2 compared with MERS-CoV (32–33% vs 23–25) [19,34]. Additionally, differences in specific domains of the S protein between SARS-CoV and SARS-CoV-2 might explain higher cross-reactivity of the tested IVIG against SARS-CoV-2 compared with SARS-CoV (80–90% vs 40–60%). The absence of cross-neutralization against MERS-CoV despite the cross-reactivity observed in ELISA assays suggests that these antibodies are not neutralizing. However, this does not necessarily indicate that such antibodies are not functional by another mechanism. For example, these non-neutralizing antibodies could be labeling the virion for identification by immune cells and subsequent destruction [35].

Despite the limitations of the *in vitro* nature of this study, the clinical implications of the findings are encouraging, and the results may support the use of IVIG as a therapeutic option for COVID-19. *In vitro* neutralization studies should be deemed as a partial characterization of a more complex response that can take place *in vivo* where the host’s response mechanisms can include antibody dependent cellular phagocytosis, antibody-dependent cellular cytotoxicity [36], as well as viral mechanisms such as antibody-dependent enhancement [37]. Nevertheless, positive results with the administration of IVIG (immunomodulatory dose) to counteract hyper inflammation in patients with severe COVID-19 [38] have already been reported in case studies [39,40]. IVIG use is being tested in an ongoing clinical trial [41]. Further studies looking at the functionality of these antibodies could improve our understanding the human coronavirus acquired immunity. This could pave the way for IVIG (and other IgG products such as intramuscular or subcutaneous preparations) as a potential therapeutic/prophylactic approach to fight current and future epidemics due to emerging HCoVs.

## Conclusion

Under the experimental conditions of this study, Flebogamma^®^ DIF and Gamunex^®^-C IVIG contained antibodies with significant neutralization capacity against SARS-CoV and SARS-CoV-2, but not against MERS-CoV. Additional research is warranted to advance IVIG toward clinical use for COVID-19.

**Summary points**

- Intravenous immunoglobulin products were tested against severe acute respiratory syndrome coronavirus 2 in cell culture neutralization assays.
- For plaque forming unit method, viral neutralization ranged from 79 to 89.5%; PRNT_50_ titers ranged from 4.5 to >5.
- For cytopathic method, viral neutralization ranged from 47 to 64.7%; IC_50_ was around 1.
- There was also neutralization of SARS-CoV, ranging from 39.5 to 55.1%; PRNT_50_; 2.0–3.3.
- Results support current trials assessing intravenous immunoglobulin as potential therapy for COVID-19.
